# Dietary antioxidants and nutritional risk in the elderly: insights from the composite dietary antioxidant index and geriatric nutritional risk index

**DOI:** 10.3389/fimmu.2025.1596663

**Published:** 2025-06-10

**Authors:** Zhongxin Zhu, Yueyuan Han, Huafang Chen

**Affiliations:** ^1^ Clinical Research Center, The First People’s Hospital of Xiaoshan District, Xiaoshan Affiliated Hospital of Wenzhou Medical University, Hangzhou, China; ^2^ The Office of Drug Clinical Trial Institution, The First Affiliated Hospital of Wenzhou Medical University, Wenzhou, China

**Keywords:** dietary intake, nutritional risk, aged, antioxidant, nutrition survey

## Abstract

**Introduction:**

The global increase in the elderly population has heightened the need to address nutritional risks in this vulnerable group. However, the relationship between overall dietary antioxidant intake and nutritional risk in the elderly remains unclear. This study aimed to investigate this association using the composite dietary antioxidant index (CDAI) and the geriatric nutritional risk index (GNRI).

**Methods:**

We analyzed data from the National Health and Nutrition Examination Survey (2010–2018), focusing on 4,208 participants aged ≥65 years. CDAI was calculated based on the intake of vitamins A, C, E, selenium, zinc, and carotenoids, while GNRI was derived from serum albumin and body weight. Multivariate regression models were employed to assess associations between CDAI, individual dietary antioxidants, and GNRI. Smooth curve fitting and two-piecewise linear regression were further performed to identify the non-linear relationships and determine the corresponding inflection points.

**Results:**

A statistically significant positive correlation was observed between the CDAI and GNRI, indicating that increased dietary antioxidant intake is linked to reduced nutritional risk. Vitamin C, selenium, zinc, and carotenoids were strongly associated with higher GNRI scores, with vitamin C and zinc showing the most robust effects. Subgroup analyses further revealed that men, diabetic individuals, and those without cancer exhibited greater improvements in nutritional risk with higher CDAI levels. Threshold effect analysis identified an optimal range for CDAI, beyond which the nutritional benefits diminished.

**Conclusions:**

Our findings highlight the critical role of dietary antioxidants, especially vitamin C and zinc, in mitigating nutritional risk among the elderly. These results support the importance of balanced dietary intake of antioxidants to optimize nutritional health in aging populations.

## Introduction

The global demographic shift towards an aging population presents significant health challenges, particularly in the realm of nutrition (1). Elderly individuals are at increased risk of nutritional deficiencies caused by reduced appetite, impaired nutrient absorption, and chronic diseases, which in turn exacerbate age-related conditions (2, 3). Consequently, addressing nutritional risks in the elderly has become a critical public health priority.

Recent studies have underscored the heterogeneous nature of the elderly population, classifying individuals into subgroups such as healthy, pre-frail, and frail, each exhibiting distinct nutritional requirements and metabolic profiles (4, 5). Among these subgroups, frail elderly individuals demonstrate heightened susceptibility to oxidative stress and chronic inflammation, factors that exacerbate nutritional decline (4, 5). Oxidative stress arises from an imbalance between reactive oxygen species and endogenous antioxidant defenses, a process that not only accelerates aging but also contributes to the pathogenesis of age-related diseases (6, 7). Dietary antioxidants have been demonstrated to mitigate oxidative stress, thereby potentially reducing the risk of chronic diseases in elderly populations (8). Given their protective effects, dietary antioxidants may serve as a vital component in strategies aimed at improving nutritional status and overall health in aging populations.

The geriatric nutritional risk index (GNRI) is a widely used tool for assessing nutritional risk in the elderly, incorporating parameters such as serum albumin levels and body weight (9). While GNRI has proven useful in predicting morbidity and mortality in older adults (10, 11), it has limitations, including its reliance on static measures that may not fully capture the dynamic nature of nutritional status. There is a growing need for more robust indicators that can provide a holistic assessment of nutritional risk, particularly in relation to dietary factors.

The composite dietary antioxidant index (CDAI) is a novel measure that aggregates the intake of multiple dietary antioxidants, providing a comprehensive assessment of antioxidant exposure (12). By considering the combined effects of various antioxidants, CDAI offers a more nuanced understanding of their role in mitigating nutritional risk. This study leverages CDAI to explore the relationship between dietary antioxidants and nutritional status in the elderly, addressing a critical gap in the existing literature.

## Methods

### Study design and population

This research analyzes data obtained from the National Health and Nutrition Examination Survey (NHANES), which is a nationally representative study administered by the National Center for Health Statistics (NCHS) to evaluate the health and nutritional status of the U.S. population. Our analysis specifically examines NHANES data collected between 2010 and 2018, providing a comprehensive and up-to-date dataset. All protocols from NHANES were approved by the NCHS Research Ethics Review Board, and informed consent was secured in writing from each participant.

**Figure 1:**
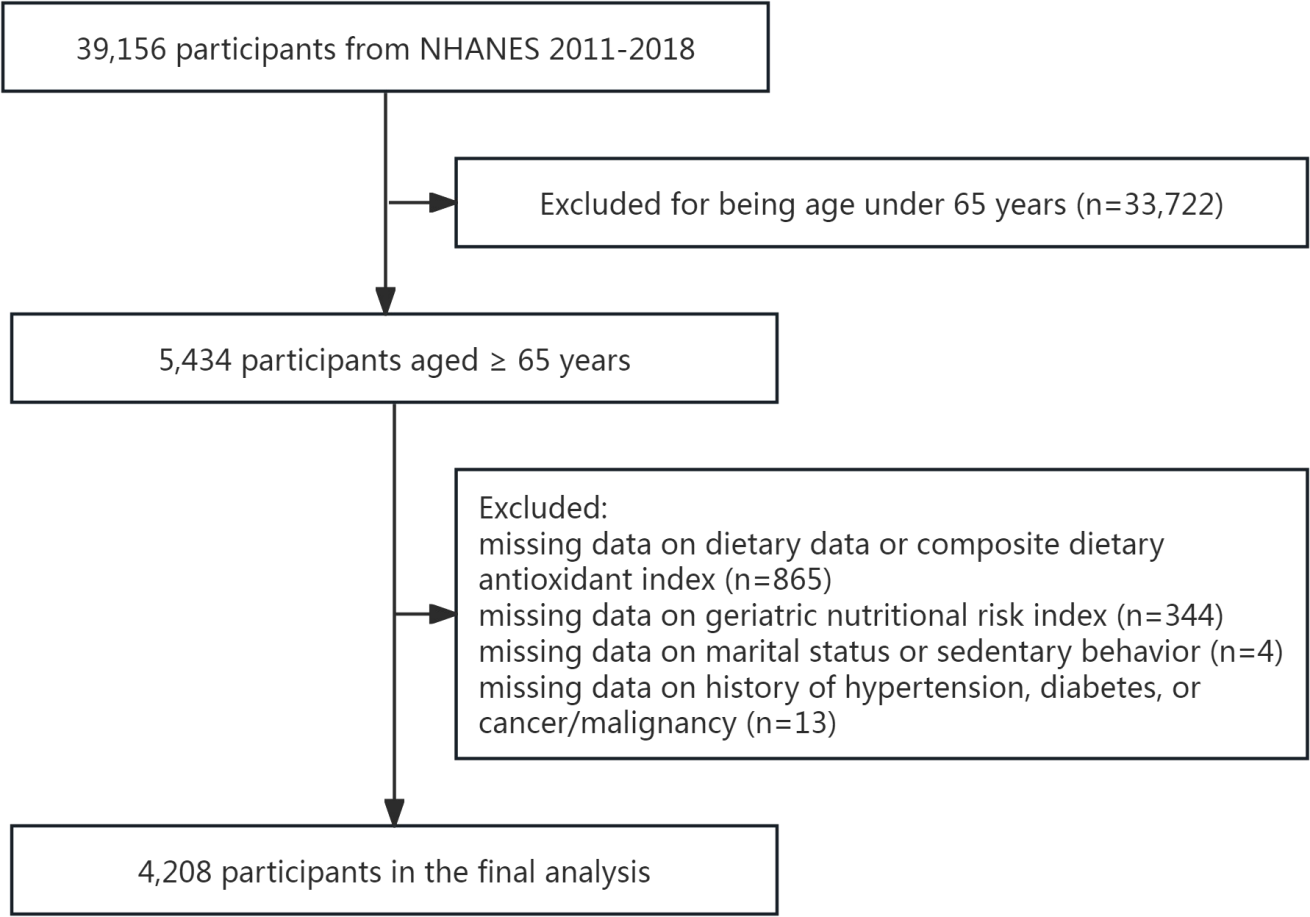
The flowchart of the participants selection.

Initially, 5,434 participants aged ≥65 years were included. After applying exclusion criteria—removing individuals with incomplete data for calculating CDAI (n=865) or GNRI (n=344), missing marital status (n=3), undefined sedentary behavior (n=1), or undocumented histories of hypertension, diabetes, or cancer (n=13)—the final analysis included 4,208 elderly subjects (**Figure 1**).

### CDAI measurement

The nutritional assessment in NHANES utilized a 24-hour dietary recall method, conducted at mobile examination centers over two non-consecutive days, with the first interview followed by a telephone interview conducted 3 to 10 days later. To reduce bias and improve accuracy, the nutrient intake for each individual was calculated as the average of the two days, compensating for any missing data from one day by using values from the other.

The CDAI was computed using a modified methodology adapted from Wright et al. (13), which incorporates six dietary antioxidants: vitamins A, C, and E, selenium, zinc, and carotenoids. The carotenoid component comprised α-carotene, lycopene, β-carotene, β-cryptoxanthin, and lutein with zeaxanthin. In alignment with prior research (14, 15), the CDAI composite score was calculated by summing the standardized values of individual micronutrients, obtained through mean-centering and scaling by their respective standard deviations. The calculation adheres to the formula: CDAI=∑ (each intake - mean value)/SD (n = 6).

### GNRI measurement

The GNRI was computed using the formula: GNRI = [1.489 × serum albumin (g/L)] + [41.7 × (body weight (kg)/ideal body weight (kg))], where ideal body weight is defined as 22 times the square of the height in meters. If the ratio of actual weight to ideal weight surpasses 1, it is normalized to 1.

### Covariates

Selected covariates were based on previous literature and clinical experience, including: (1). demographic data: age, sex, race/ethnicity (non-Hispanic White, non-Hispanic Black, Mexican American, other race/ethnicity), education level (less than high school, high school, more than high school), marital status (married/living with partner, widowed/divorced/separated, never married), and poverty income ratio (categorized as low [<1.3], medium [1.3–3.5], or high [>3.5]); (2). examination data: body mass index; (3). questionnaire data: sedentary behavior (defined as the absence of moderate or vigorous physical activity) and history of hypertension, diabetes, and cancer//malignancy. Demographic data were collected through in-home interviews, examination data were measured at mobile examination centers, and questionnaire data were obtained via self-reported conditions.

### Statistical analyses

The baseline characteristics of the subjects were categorized according to the quartiles of the CDAI. Continuous variables are reported as mean ± standard deviation, whereas categorical variables are expressed as percentages. To assess differences between groups, we utilized χ² tests for categorical data, one-way ANOVA for normally distributed continuous data, and Kruskal-Wallis H tests for data with skewed distributions.

To examine the associations between CDAI, individual dietary antioxidants, and GNRI, we implemented a tiered multivariate linear regression analysis following the STrengthening the Reporting of OBservational studies in Epidemiology (STROBE) guidelines (16). Three sequential models were constructed: Model 1 (crude): unadjusted analysis to assess raw associations; Model 2 (partially adjusted): controlled for core demographic confounders (age, sex, race/ethnicity); and Model 3 (fully adjusted): further adjusted for socioeconomic, lifestyle, and clinical covariates. Subgroup analyses assessed effect modification by stratifying key covariates, with multiplicative interaction terms tested for statistical significance. To address non-linearity, we applied: generalized additive models with smoothing splines, which flexibly capture non-parametric trends without presupposing functional forms; and two-piecewise linear regression to objectively identify inflection points where the relationship shifted. This method was favored over polynomial regression due to its clinical utility in defining actionable thresholds.

Statistical analyses were carried out using R software (version 3.4.3) and EmpowerStats (X&Y Solutions, Inc., Boston, MA). A two-sided P value of less than 0.05 was regarded as statistically significant.

## Results

### Study population characteristics

The characteristics of the study population categorized by quartiles of CDAI are detailed in **Table 1**. A total of 4,208 participants were divided into four quartiles, revealing significant disparities in demographic and health-related factors among the groups. The percentage of men increased from 36.6% in Q1 to 61.9% in Q4, while the proportion of women decreased correspondingly. Individuals with education beyond high school increased from 36.3% in Q1 to 65.1% in Q4. The frequency of sedentary behavior decreased from 70.5% in Q1 to 50.8% in Q4. The prevalence of hypertension, diabetes, and cancer showed an inverse relationship with CDAI quartiles. Additionally, dietary assessments indicated a significant rise in both energy and antioxidants intake, while GNRI demonstrated a slight increase across the quartiles.

**Table 1:** Characteristics of study population based on composite dietary antioxidant index quartiles.

Composite dietary antioxidant index	Q1 (≤-2.575)	Q2 (-2.573 to -0.580)	Q3 (-0.579 to 2.017)	Q4 (≥2.018)	P value
Age (years)	72.9 ± 5.3	73.3 ± 5.4	73.3 ± 5.3	73.0 ± 5.4	0.208
Sex, n (%)					<0.001
Men	385 (36.6%)	485 (46.1%)	578 (54.9%)	651 (61.9%)	
Women	667 (63.4%)	567 (53.9%)	474 (45.1%)	401 (38.1%)	
Race/Ethnicity, n (%)					<0.001
Non-Hispanic White	449 (42.7%)	511 (48.6%)	613 (58.3%)	609 (57.9%)	
Non-Hispanic Black	268 (25.5%)	209 (19.9%)	167 (15.9%)	164 (15.6%)	
Mexican American	108 (10.3%)	121 (11.5%)	92 (8.7%)	82 (7.8%)	
Other race/ethnicity	227 (21.6%)	211 (20.1%)	180 (17.1%)	197 (18.7%)	
Education level, n (%)					<0.001
Less than high school	409 (38.9%)	310 (29.5%)	256 (24.3%)	170 (16.2%)	
High school	261 (24.8%)	262 (24.9%)	249 (23.7%)	197 (18.7%)	
More than high school	382 (36.3%)	480 (45.6%)	547 (52.0%)	685 (65.1%)	
Marital status, n (%)					<0.001
Married/Living with partner	503 (47.8%)	561 (53.3%)	637 (60.6%)	653 (62.1%)	
Widowed/Divorced/Separated	490 (46.6%)	445 (42.3%)	377 (35.8%)	350 (33.3%)	
Never married	59 (5.6%)	46 (4.4%)	38 (3.6%)	49 (4.7%)	
Poverty income ratio, n (%)					<0.001
Low	354 (33.7%)	282 (26.8%)	256 (24.3%)	196 (18.6%)	
Medium	402 (38.2%)	432 (41.1%)	426 (40.5%)	400 (38.0%)	
High	171 (16.3%)	220 (20.9%)	276 (26.2%)	357 (33.9%)	
Unrecorded	125 (11.9%)	118 (11.2%)	94 (8.9%)	99 (9.4%)	
Sedentary behavior, n (%)					<0.001
Yes	742 (70.5%)	687 (65.3%)	610 (58.0%)	534 (50.8%)	
No	310 (29.5%)	365 (34.7%)	442 (42.0%)	518 (49.2%)	
Hypertension, n (%)					<0.001
Yes	708 (67.3%)	697 (66.3%)	646 (61.4%)	644 (61.2%)	
No	344 (32.7%)	355 (33.7%)	406 (38.6%)	408 (38.8%)	
Diabetes, n (%)					<0.001
Yes	305 (29.0%)	307 (29.2%)	272 (25.9%)	233 (22.1%)	
No	747 (71.0%)	745 (70.8%)	780 (74.1%)	819 (77.9%)	
Cancer/malignancy, n (%)					<0.001
Yes	209 (19.9%)	250 (23.8%)	266 (25.3%)	298 (28.3%)	
No	843 (80.1%)	802 (76.2%)	786 (74.7%)	754 (71.7%)	
Body mass index (kg/m^2^)	29.3 ± 6.3	29.0 ± 6.0	29.2 ± 6.2	28.5 ± 5.8	0.005
Dietary energy (kcal)	1181.6 ± 391.8	1633.9 ± 430.0	1955.3 ± 506.0	2374.7 ± 764.4	<0.001
Dietary vitamin A (mcg)	294.1 ± 162.2	489.6 ± 215.7	670.4 ± 285.2	1156.7 ± 936.1	<0.001
Dietary vitamin C (mg)	36.2 ± 31.9	63.1 ± 42.9	87.0 ± 54.3	139.8 ± 86.2	<0.001
Dietary vitamin E (mg)	4.1 ± 1.8	6.2 ± 2.4	8.3 ± 3.0	12.9 ± 6.7	<0.001
Dietary selenium (mcg)	62.7 ± 22.1	88.0 ± 26.4	109.2 ± 32.5	138.1 ± 55.9	<0.001
Dietary zinc (mg)	5.6 ± 2.1	8.4 ± 2.5	10.6 ± 3.2	14.6 ± 6.5	<0.001
Dietary carotenoids (mg)	3.0 ± 2.8	5.7 ± 4.2	9.0 ± 6.0	17.0 ± 12.8	<0.001
Geriatric nutritional risk index	102.5 ± 5.0	102.8 ± 5.1	103.4 ± 5.0	103.5 ± 4.6	<0.001

### Associations between antioxidants and GNRI

**Figure 2:**
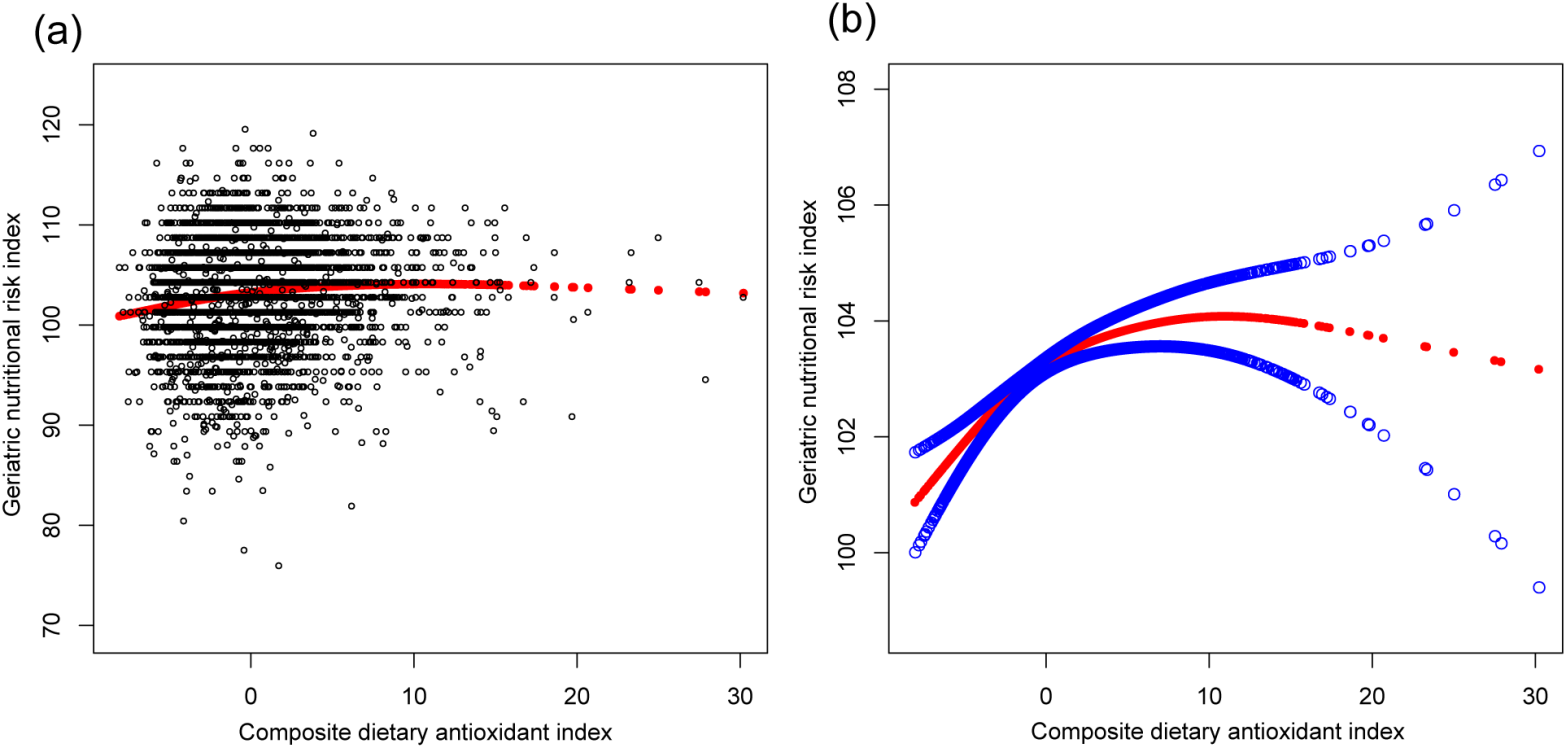
The association between composite dietary antioxidant index and geriatric nutritional risk index. **(a)** Each black point represents a sample. **(b)** Solid red line represents the smooth curve fit between variables. Blue bands represent the 95% of confidence interval from the fit. Age, sex, race, education level, marital status, poverty income ratio, sedentary behavior, body mass index, dietary energy, history of hypertension, diabetes, and cancer were adjusted.

**Figure 3:**
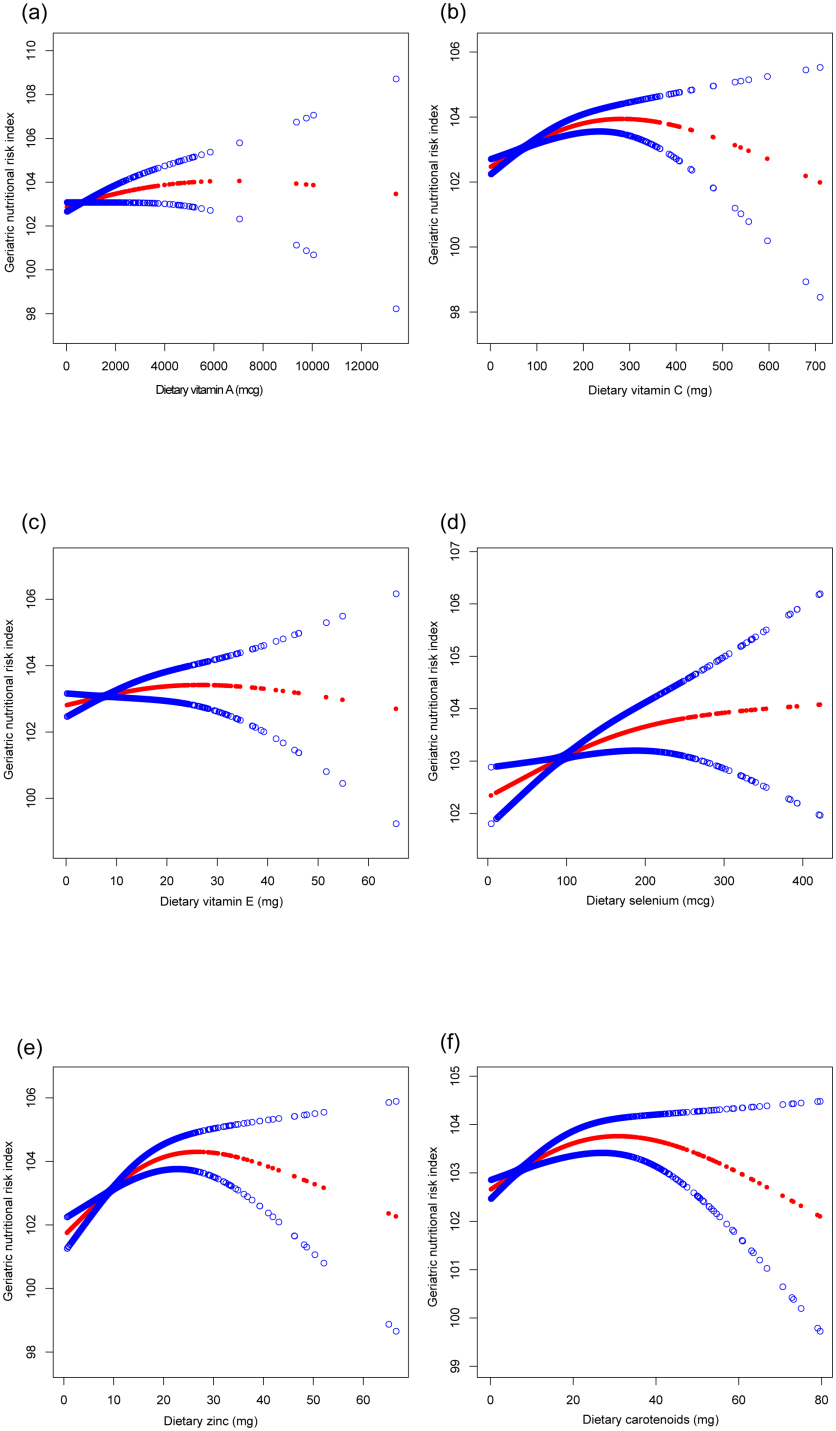
The association between six dietary antioxidants intake and geriatric nutritional risk index. **(a)** Dietary vitamin A; **(b)** Dietary vitamin C; **(c)** Dietary vitamin E; **(d)** Dietary selenium; **(e)** Dietary zinc; **(f)** Dietary carotenoids. Age, sex, race, education level, marital status, poverty income ratio, sedentary behavior, body mass index, dietary energy, history of hypertension, diabetes, and cancer were adjusted.

**Table 2** presents the associations between the CDAI, individual dietary antioxidants, and the GNRI. The CDAI exhibited a significant positive correlation with the GNRI across all models (Model 3: β=0.121, 95% CI: 0.072, 0.171). Dietary intake of vitamin C, selenium, zinc, and carotenoids was positively associated with the GNRI, particularly for vitamin C (Model 3: β=0.005, 95% CI: 0.003, 0.007) and zinc (Model 3: β=0.076, 95% CI: 0.039, 0.113). These relationships, along with potential non-linear trends, are illustrated in **Figures 2**, **3**.

**Table 2:** Association between composite dietary antioxidant index, six dietary antioxidants and geriatric nutritional risk index.

Model 1	β (95% CI), Model 2	β (95% CI), Model 3	β (95% CI)
Composite dietary antioxidant index	0.094 (0.057, 0.132) ^***^	0.059 (0.021, 0.097) ^**^	0.121 (0.072, 0.171) ^***^
Q1	Reference	Reference	Reference
Q2	0.330 (-0.091, 0.750)	0.219 (-0.197, 0.634)	0.493 (0.065, 0.922)
Q3	0.946 (0.525, 1.366)	0.704 (0.284, 1.124)	1.138 (0.674, 1.602)
Q4	1.059 (0.638, 1.480)	0.709 (0.285, 1.133)	1.332 (0.802, 1.862)
P for trend	<0.001	<0.001	<0.001
Dietary vitamin A (mcg)	0.000 (-0.000, 0.000)	0.000 (-0.000, 0.000)	0.000 (-0.000, 0.000)
Dietary vitamin C (mg)	0.006 (0.004, 0.008) ^***^	0.005 (0.003, 0.007) ^***^	0.005 (0.003, 0.007) ^***^
Dietary vitamin E (mg)	0.027 (-0.002, 0.056)	0.008 (-0.021, 0.037)	0.019 (-0.016, 0.054)
Dietary selenium (mcg)	0.005 (0.002, 0.009) ^**^	0.000 (-0.003, 0.004)	0.006 (0.001, 0.010) ^*^
Dietary zinc (mg)	0.066 (0.037, 0.095) ^***^	0.031 (0.001, 0.061) ^*^	0.076 (0.039, 0.113) ^***^
Dietary carotenoids (mg)	0.034 (0.017, 0.050) ^***^	0.028 (0.012, 0.044) ^***^	0.026 (0.010, 0.043) ^**^

Model 1: no covariates were adjusted.

Model 2: age, sex and race were adjusted.

Model 3: age, sex, race, education level, marital status, poverty income ratio, sedentary behavior, body mass index, dietary energy, history of hypertension, diabetes, and cancer were adjusted.

^*^P <0.05, ^**^P <0.01, ^***^P <0.001.

### Subgroup analysis

**Figure 4:**
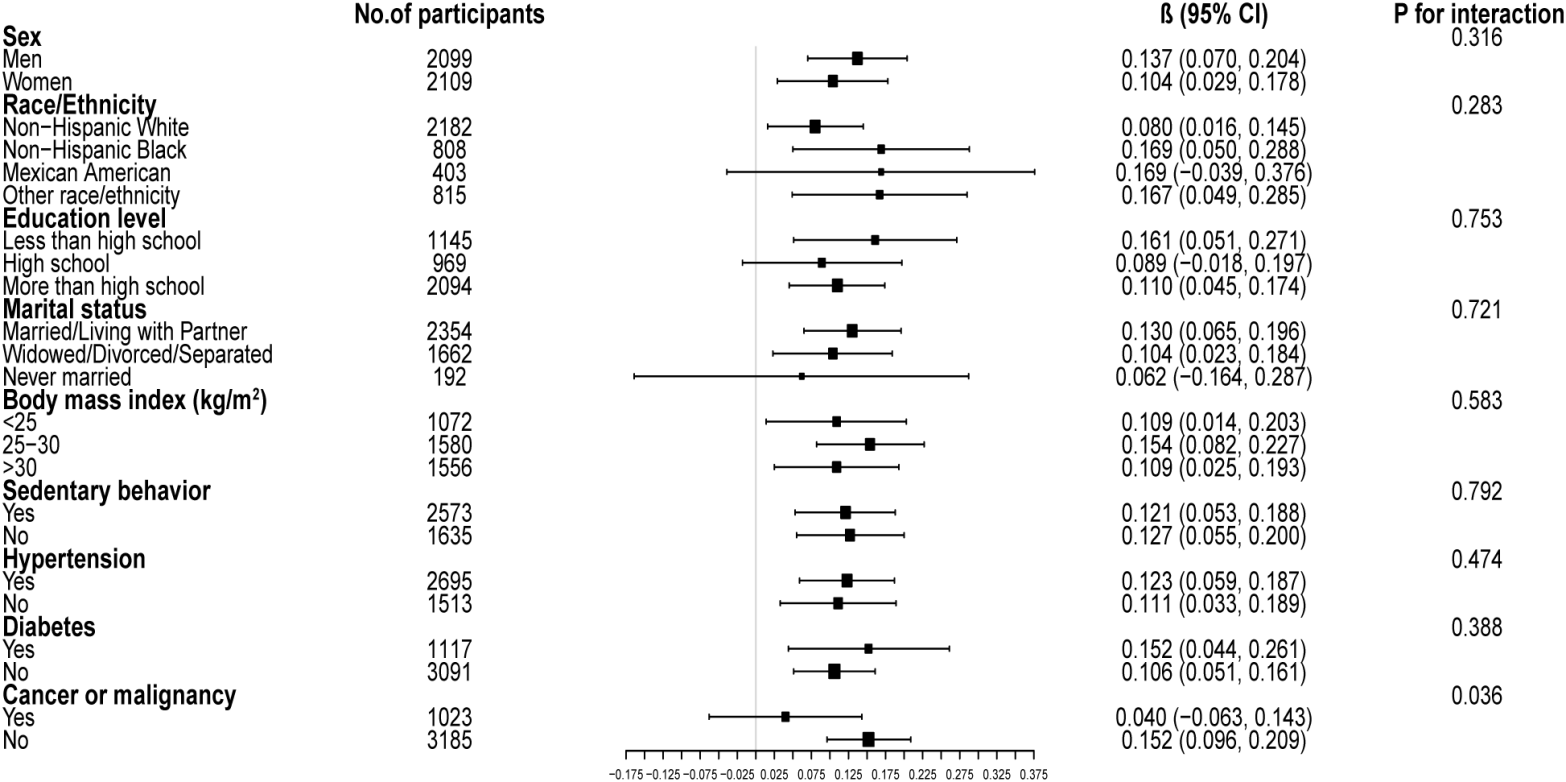
Subgroup analysis of the associations between composite dietary antioxidant index and geriatric nutritional risk index. Age, sex, race, education level, marital status, poverty income ratio, sedentary behavior, body mass index, dietary energy, history of hypertension, diabetes, and cancer were adjusted. In the subgroup analysis, the model is not adjusted for the stratification variable itself.

**Figure 5:**
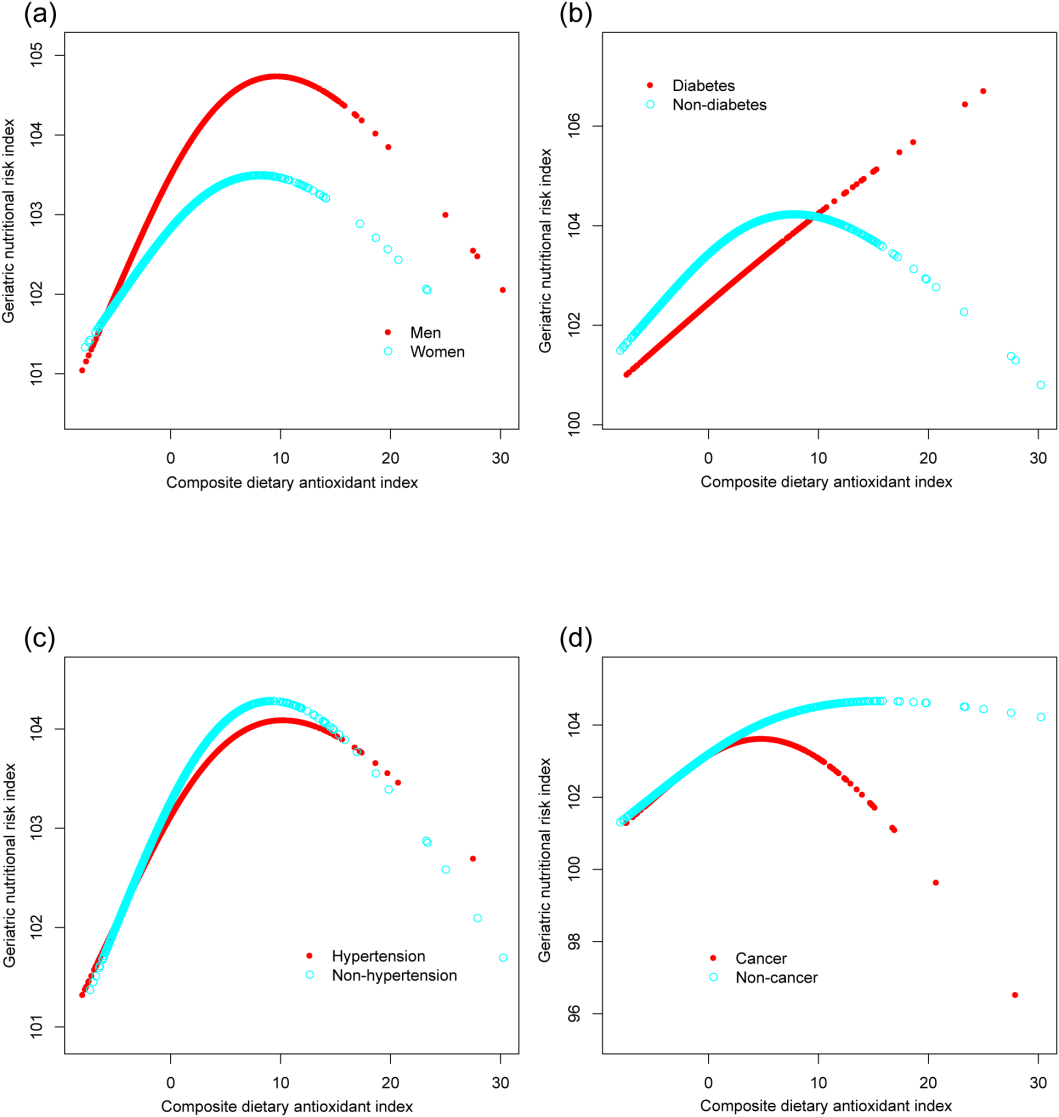
The associations between composite dietary antioxidant index and geriatric nutritional risk index, stratified by sex **(a)**, history of diabetes **(b)**, hypertension **(c)**, and cancer **(d)**. Age, sex, race, education level, marital status, poverty income ratio, sedentary behavior, body mass index, dietary energy, history of hypertension, diabetes, and cancer were adjusted. In the subgroup analysis, the model is not adjusted for the stratification variable itself.

**Figure 4** illustrates the subgroup analysis concerning the CDAI and the GNRI. The β coefficient for men was higher (0.137, 95% CI: 0.070, 0.204) compared to women (0.104, 95% CI: 0.029, 0.178). Participants with diabetes had a β coefficient of 0.152 (95% CI: 0.044, 0.261), greater than that of non-diabetic individuals (β=0.106, 95% CI: 0.051, 0.161). Participants without cancer presented a significantly elevated β coefficient (0.152, 95% CI: 0.096, 0.209) compared to those with cancer (β=0.040, 95% CI: -0.063, 0.143). Potential non-linear relationships stratified by sex, history of hypertension, diabetes, and cancer are further confirmed in **Figure 5**.

### Threshold effect analysis

Analysis of the threshold effect of CDAI on GNRI revealed distinct patterns across various subgroups (**Table 3**). For men, the inflection point was determined at 10, indicating a significant positive association below this threshold (β=0.201, 95% CI: 0.122, 0.279) and a non-significant negative association above it (β=-0.177, 95% CI: -0.394, 0.040). Women exhibited a similar trend, with an inflection point at 8.5. Participants without diabetes showed a significant positive association when the CDAI was below 8.5 (β=0.167, 95% CI: 0.102, 0.232), whereas those with cancer displayed a notable inflection point at 5, with significant associations observed both below and above this threshold.

**Table 3:** Threshold effect analysis of composite dietary antioxidant index on geriatric nutritional risk index using two-piecewise linear regression model.

Geriatric nutritional risk index	Adjusted ß (95% CI), p-value
Men	
Inflection point	10
Composite dietary antioxidant index <10	0.201 (0.122, 0.279), <0.001
Composite dietary antioxidant index >10	-0.177 (-0.394, 0.040), 0.109
Log likelihood ratio	0.003
Women	
Inflection point	8.5
Composite dietary antioxidant index <8.5	0.143 (0.058, 0.227), 0.001
Composite dietary antioxidant index >8.5	-0.158 (-0.444, 0.127), 0.277
Log likelihood ratio	0.061
Participants without diabetes	
Inflection point	8.5
Composite dietary antioxidant index <8.5	0.167 (0.102, 0.232), <0.001
Composite dietary antioxidant index >8.5	-0.177 (-0.346, -0.008), 0.040
Log likelihood ratio	<0.001
Participants with hypertension	
Inflection point	10
Composite dietary antioxidant index <10	0.159 (0.087, 0.231), <0.001
Composite dietary antioxidant index >10	-0.196 (-0.490, 0.098), 0.192
Log likelihood ratio	0.029
Participants without hypertension	
Inflection point	9.5
Composite dietary antioxidant index <9.5	0.175 (0.081, 0.268), <0.001
Composite dietary antioxidant index >9.5	-0.137 (-0.355, 0.082), 0.220
Log likelihood ratio	0.017
Participants with cancer	
Inflection point	5
Composite dietary antioxidant index <5	0.209 (0.069, 0.349), 0.003
Composite dietary antioxidant index >5	-0.279 (-0.485, -0.072), 0.008
Log likelihood ratio	<0.001

Age, sex, race, education level, marital status, poverty income ratio, sedentary behavior, body mass index, dietary energy, history of hypertension, diabetes, and cancer were adjusted. In the subgroup analysis, the model is not adjusted for the stratification variable itself.

## Discussion

Our study demonstrates a significant positive association between CDAI and GNRI, indicating that higher dietary antioxidant intake is linked to reduced nutritional risk in elderly individuals. Specifically, vitamin C, selenium, zinc, and carotenoids showed strong positive associations with GNRI, with vitamin C and zinc exhibiting the most pronounced effects. These findings highlight the critical role of dietary antioxidants in mitigating nutritional risk in aging populations.

The CDAI-GNRI correlation demonstrates that dietary antioxidants mitigate age-related nutritional decline. This effect may occur through dual mechanisms of reducing oxidative stress and inflammation, which are key drivers of nutritional decline that impair cellular function and exacerbate muscle wasting, ultimately helping preserve nutritional status in elderly populations (17–19). The robust associations observed for vitamin C and zinc may be attributed to their essential roles in immune function and cellular repair. Vitamin C plays a crucial role in cellular antioxidant defense by scavenging free radicals in the aqueous phase, thereby preventing oxidative damage to proteins, lipids, and carbohydrates. Additionally, it contributes to the regeneration of vitamin E, which is vital for maintaining the integrity of the cellular antioxidant network (20, 21). Zinc, on the other hand, is crucial for decreased oxidative stress biomarkers and decreased inflammatory cytokines in the elderly, and its deficiency is closely linked to malnutrition in the elderly (22, 23). The differential effects of antioxidants may also reflect variations in bioavailability and synergistic interactions. For instance, carotenoids, being fat-soluble, are better absorbed in the presence of dietary fats, whereas vitamin C, being water-soluble, is more readily excreted (24, 25).

Our findings are consistent with prior studies highlighting the beneficial effects of dietary antioxidants on health outcomes in the elderly, a population particularly vulnerable to nutrition-related risks. Evidence highlights their protective effects against cardiovascular diseases, with studies showing that high antioxidants intake reduces myocardial infarction risk (26). Antioxidants also support cognitive health, with plant foods rich in antioxidants linked to significant beneficial effects on cognitive functions risk (27, 28). Additionally, antioxidants counteract osteoporosis by mitigating oxidative damage, potentially accelerating fracture healing (29). Furthermore, they contribute to cancer prevention by neutralizing oxidative stress, a key pathogenic factor in carcinogenesis, which is particularly relevant for the elderly due to cumulative lifetime exposure (30). Our threshold analysis identified distinct optimal ranges for antioxidant intake, with CDAI values below 10.0 in men and 8.5 in women demonstrating maximal nutritional benefits. These gender-specific inflection points may reflect fundamental differences in body composition, oxidative stress response thresholds, and nutrient utilization efficiency. These findings suggest that geriatric nutritional interventions should incorporate sex-specific antioxidant recommendations to optimize therapeutic outcomes.

This study advances current knowledge by introducing CDAI as a novel tool for assessing dietary antioxidant intake and its relationship with nutritional risk. The observed inverted U-shaped relationship between antioxidant intake and GNRI may be attributed to nutrient saturation and the pro-oxidant effects of high doses of certain antioxidants. The concept of nutrient saturation indicates that antioxidant benefits peak at specific intake levels, beyond which additional consumption yields no further advantages and may pose potential risks (31, 32). High doses of certain antioxidants, such as vitamin C, can exhibit pro-oxidant activity, increasing oxidative stress and potentially damaging cellular components, including DNA and proteins (33, 34). Our findings support the important role of dietary antioxidants in reducing nutritional risk among elderly populations. Specifically, vitamin C-rich foods such as citrus fruits, berries, kiwi and bell peppers (33), zinc-rich foods including legumes, seafood, lean meats and fortified cereals (35), and balanced dietary patterns like the Mediterranean diet (36) may collectively help mitigate age-related nutritional deficiencies. These findings underscore the need for public health initiatives to develop tailored dietary guidelines for the elderly, prioritizing a varied intake of antioxidant-rich whole foods to optimize nutritional risk reduction. Importantly, given the observed threshold effects and potential pro-oxidant risks associated with high-dose supplementation, excessive isolated antioxidant intake should be discouraged. To facilitate implementation, clinicians and policymakers could incorporate CDAI-based screening into routine geriatric nutritional assessments, enabling early identification of individuals with suboptimal antioxidant intake and guiding personalized dietary interventions.

To the best of our knowledge, this study represents the first investigation into the relationship between CDAI and GNRI within a nationally representative sample of elderly individuals. However, several limitations should be acknowledged. First, the NHANES database predominantly utilizes a cross-sectional design, which inherently restricts the ability to infer causality. While our findings demonstrate a significant association between antioxidants intake and nutritional risk, they do not establish whether increased antioxidant consumption directly reduces nutritional risk. The observed association between higher antioxidant intake and reduced nutritional risk may be influenced by reverse causality, wherein individuals with better baseline nutritional status are more likely to consume antioxidant-rich foods. To elucidate the causal relationship and temporal dynamics, future longitudinal cohort studies or randomized controlled trials are warranted. Second, dietary data in NHANES are collected through 24-hour dietary recalls, a method susceptible to recall bias. This limitation is particularly relevant for elderly participants, who may experience memory decline or cognitive impairments, potentially leading to inaccuracies in reporting dietary intake. Underreporting or overreporting of specific antioxidants may introduce measurement bias into the CDAI calculation, thereby either attenuating or inflating the observed associations. Third, although the study adjusted for a range of covariates, residual confounding may persist due to unmeasured variables such as genetic predispositions, environmental exposures, cognitive assessment or the severity of chronic diseases, which could influence the observed associations. For instance, cognitive impairment, a prevalent condition among the elderly yet unmeasured in our analysis, may systematically influence dietary recall accuracy, introducing potential confounding. Furthermore, the severity of underlying chronic conditions could act as an effect modifier in the antioxidant-GNRI relationship. These limitations underscore the necessity for future studies incorporating multidimensional clinical evaluations to validate and refine these observational associations.

## Conclusion

In summary, this study underscores the critical role of dietary antioxidants in reducing nutritional risk among elderly individuals. The robust positive association between CDAI and GNRI, particularly driven by vitamin C and zinc, highlights the potential of these nutrients to enhance nutritional health in aging populations. The observed inverted U-shaped relationship between antioxidants intake and GNRI suggests that while moderate consumption offers significant protective benefits, excessive intake may result in diminishing returns or adverse effects. These findings advocate for a balanced dietary approach, emphasizing diverse food intake to achieve optimal antioxidant levels and improve nutritional outcomes, particularly in elderly populations at higher risk of deficiencies.

## Data Availability

The datasets presented in this study can be found in online repositories. The names of the repository/repositories and accession number(s) can be found below: https://wwwn.cdc.gov/nchs/nhanes/Default.aspx.
